# Understanding the effects of a complex psychological intervention on symptoms of depression in Goa, India: findings from a causal mediation analysis

**DOI:** 10.1192/bjp.2022.116

**Published:** 2023-02

**Authors:** Nadine Seward, Stijn Vansteelandt, Darío Moreno-Agostino, Vikram Patel, Ricardo Araya

**Affiliations:** Centre for Global Mental Health, Health Service and Population Research Department, Institute of Psychiatry, Psychology & Neuroscience, King's College London, UK; and Centre for Implementation Science, Health Service and Population Research Department, Institute of Psychiatry, Psychology & Neuroscience, King's College London, UK; Department of Applied Mathematics, Computer Science and Statistics, Ghent University, Belgium; and Department of Medical Statistics, London School of Hygiene and Tropical Medicine, UK; Centre for Global Mental Health, Health Service and Population Research Department, Institute of Psychiatry, Psychology & Neuroscience, King's College London, UK; and Centre for Longitudinal Studies, UCL Social Research Institute, University College London, UK; Department of Global Health and Social Medicine, Harvard Medical School, Massachusetts, USA; Centre for Global Mental Health, Health Service and Population Research Department, Institute of Psychiatry, Psychology & Neuroscience, King's College London, UK

**Keywords:** Low- and middle-income countries, psychosocial interventions, depressive disorders, task-sharing, behavioural activation

## Abstract

**Background:**

Understanding how and under what circumstances a highly effective psychological intervention, improved symptoms of depression is important to maximise its clinical effectiveness.

**Aims:**

To address this complexity, we estimate the indirect effects of potentially important mediators to improve symptoms of depression (measured with the Patient Health Questionnaire (PHQ-9)) in the Healthy Activity Program trial.

**Method:**

Interventional in(direct) effects were used to decompose the total effect of the intervention on PHQ-9 scores into the direct and indirect effects. The following indirect effects were considered: characteristics of sessions, represented by the number of sessions and homework completed; behavioural activation, according to an adapted version of the Behavioural Activation for Depression Scale – Short Form; and extra sessions offered to participants who did not respond to the intervention.

**Results:**

Of the total effect of the intervention measured through the difference in PHQ-9 scores between treatment arms (mean difference: −2.1, bias-corrected 95% CI −3.2 to −1.5), 34% was mediated through improved levels of behavioural activation (mean difference: −0.7, bias-corrected 95% CI −1.2 to −0.4). There was no evidence to support the mediating role of characteristics of the sessions nor the extra sessions offered to participants who did not respond to the treatment.

**Conclusions:**

Findings from our robust mediation analyses confirmed the importance of targeting behavioural activation. Contrary to published literature, our findings suggest that neither the number of sessions nor proportion of homework completed improved outcomes. Moreover, in this context, alternative treatments other than extra sessions should be considered for patients who do not respond to the intervention.

Depression is a common mental health disorder that affects an estimated 300 million people worldwide and is the leading mental health contributor to the global burden of disease.^1^ Depression is also associated with excess mortality and morbidity, as well as profound social and economic consequences. Despite this, ≤10% of people with depression in low- and middle-income countries (LMICs) have access to effective treatment.

Psychological treatments are the recommended first-line intervention for depression according to the World Health Organization's Mental Health Gap Action Programme, as they have been shown to be just as effective as pharmacological interventions and have a sustained effect over time.^2^ However, important barriers to accessing these treatments in LMICs exist, in particular the lack of trained professionals.^3^ This has led to the design of briefer treatments and task-sharing interventions to trained non-specialists.^4–7^ Trials using different therapeutic approaches for depression (e.g. psychoeducation, problem-solving and behavioural activation), by way of a task-sharing modality, have demonstrated varying levels of effectiveness in LMICs.^8–10^

## The Healthy Activity Program

One of the most successful trials of a lay counsellor-delivered psychological intervention for depression from an LMIC is the Healthy Activity Program (HAP) trial.^11^ The HAP trial is an adapted form of behavioural activation delivered by lay counsellors on a face-to-face modality for participants with moderate-to-severe depression in primary care settings in Goa, India. Findings suggest that the HAP trial improved remission from depression both at the end of the trial and at 12 months’ follow-up. However, little is known surrounding how the trial improved recovery from depression, other than findings from a mediation analysis that found 58% of the total effect was mediated through improved activation, measured using the Behavioural Activation for Depression Scale – Short Form (BADS-SF).^12^

Several mediators have been identified that help to explain how the HAP intervention improved symptoms of depression. In particular, characteristics of the sessions are known to influence depression outcomes through different pathways. As an example, evidence suggests that the number of sessions is an important predictor of recovery, with a minimum of five to six sessions required to improve depression outcomes.^11,13^ Behavioural activation has been identified as an important mechanism through which symptoms of intervention improve.^14^ Analyses that account for the multiple and often interacting pathways through which an intervention operates, including both characteristics of the sessions and mechanisms such as behavioural activation, will help clinicians to understand how the intervention can be optimised to help their patients recover from depression. Comparing findings across different studies will provide insight into what works for whom and under what circumstances.

The interventional (in)direct effects method is one of the latest in a series of recent advancements to causal mediation analyses that allows for the decomposition of the total effect of a complex intervention into multiple indirect effects that can characterise specific mechanisms and characteristics of the sessions.^15^ By simultaneously including multiple mediators, their interactions and non-linearities, the interventional (in)direct effects can reduce biases that are often present in analyses that use traditional approaches. Importantly, this approach ensures that the direct and indirect effects are summed to reach the total effect. The resulting decomposition of the total effect provides insight into effects that improve outcomes, as well as effects that potentially worsen outcomes.

When using linear and additive mean models where all assumptions are fulfilled (e.g. no interactions between the different mediators), estimators of interventional indirect effects produce identical estimators to product-of-coefficient estimators, assuming a parallel mediator model.^16^ However, in practice, these modelling assumptions are rarely fulfilled. Therefore, when multiple mediation analysis is used to estimate the indirect effects of different mediators under non-linear or non-additive models, the total sum of the different direct and indirect effects does not generally equal the total effect of the intervention.

We therefore estimated interventional (in)direct effects based on data collected both at 3 and 12 months after the trial started, for the following mediators: characteristics of the sessions, including number of sessions (M1a) and proportion of homework completed (M1b); behavioural activation (M2); and whether a participant responded to therapy (M3a) and extra sessions they received in this instance (M3b). It is hoped that our findings will provide further insights into what components of the intervention worked (or not).

## Method

### Setting

We used data from both arms of a previously conducted randomised controlled trial (HAP) that took place between October 2013 and July 2015 in primary healthcare centres in Goa, India, and included information on symptoms of depression and important mediators at 3 and 12 months after the trial started.^11,12^ The original trial was registered with the ISRCTN registry, under identifier ISRCTN95149997.

### Design

The HAP study was a parallel-arm, individually randomised controlled trial with equal allocation of participants between arms. Participants aged 18–65 years were recruited from ten primary health centres. Eligibility criteria included a probable diagnosis of moderate-to-severe depression, determined by a Patient Health Questionnaire (PHQ-9) score of >14. Pregnant women and participants who needed urgent medical attention or who were unable to communicate were excluded. The HAP trial was powered to evaluate the effect of the intervention on the primary outcome, and not for the estimates calculated with this mediation analyses.

### The intervention

The intervention (HAP) was a manualised psychological intervention based on behavioural activation for depression that primarily involved strategies to increase the number of enjoyable activities a person engaged with.^11,17^ Other strategies were also included after exploring their acceptability, appropriateness and feasibility in the local context, including need-based strategies that addressed interpersonal triggers, problem-solving, relaxation and enlisting social support tailored to the specific need of the individuals.^18^

The experimental arm received up to eight sessions (described below), lasting between 30 and 40 min, at weekly intervals over a 3-month period. The sessions were usually face to face at the primary health centre, or the patient's home. Telephone sessions were used only when strictly necessary. The intervention was organised into three phases. The first phase (sessions 1 and 2) was primarily used to engage the participant; establish an effective relationship; explain the objectives of the sessions, including behavioural activation; and elicit a commitment for the HAP intervention. The middle phase (sessions 3 and 4) assessed activation targets and encouraged activation, identifying barriers to activation, and learning to overcome these and how to solve or cope with life problems. The final phase (sessions 5 and 6) reviewed and strengthened gains the patient made during treatment, so as to prevent relapse. If a participant did not respond to treatment by the third or fourth session, two additional middle-phase sessions were offered, resulting in these patients attending a total of seven to eight sessions.

The HAP intervention was delivered by lay counsellors who had completed at least the tenth grade of education and were fluent in local languages. Counsellors were also required to meet predefined competency standards.^19^ Training took place over a 2-week period. Counsellors received weekly peer-led supervision in groups and individual supervision twice a month.

Enhanced usual care (EUC) was offered to participants in both arms of the trial.^11^ EUC involved screening results for depression being shared with both patients and physician. Physicians were also trained on how to use a contextualised version of the Mental Health Gap Action Programme guidelines, including when and where to refer for psychiatric care. There were no sessions offered to participants in the control arm of the trial.

### Measures

#### Exposure

Our exposure of interest was the HAP intervention that was offered to participants in the experimental arm of the trial only.

#### Outcome

For our analysis, our primary outcome was the PHQ-9 score at 12 months. Response options generate a continuous score ranging from 0 to 27, since each of the nine items can be scored from zero (no symptoms) to three (nearly every day). Scores between 10 and 14 represent moderate depression, and scores between 15 and 27 represent moderately severe to severe depression symptoms.^20^

#### Mediators

Causal mediation analyses require obtaining estimates in both the exposed and unexposed (i.e. the counterfactual) participants. Where mediators were measured in both the experimental and control arms, estimates from participants in the control arm can be treated as the unexposed. However, where a mediator was only measured in the experimental arm, it was necessary to create a separate category for participants who had not been exposed to the mediator of interest, but were still in the experimental arm.

### Therapeutic process indicators (M1)

Characteristics associated with the delivery of the sessions were measured for participants in the experimental arm only. The first characteristic that we accounted for is the number of sessions completed (M1a), categorised to reflect the phases of the HAP intervention that a participant completed: no sessions (unexposed), sessions one and two (phase 1), sessions three and four (phase 2) and sessions five to eight (phase 3).

The second characteristic of the sessions that we accounted for is a participant's self-reported completion of assigned tasks outside of sessions (homework), to improve activity levels. At each session except for the first one, activity monitoring charts were completed indicating whether a participant completed homework outside of the sessions. These self-reported activity charts were scored with the following criteria: completely (scored 2), partially (scored 1) or not at all (scored 0). Based on this variable, we calculated a score representing the proportion of homework completed (M1b): 0, indicating none (unexposed); 1, indicating >0% to ≤50%; and 2, indicating >50%.

### Level of behavioural activation (M2)

Behavioural activation was measured for participants in both the experimental (exposed) and control arms (unexposed) at 3 months after the trial started. An adapted version of the BADS-SF was used to capture activity levels that reflect the level of behavioural activation resulting from the HAP intervention.^21^

### Extra sessions received in instances of non-response to the intervention (M3)

Adding extra sessions for participants who do not respond to the intervention may help to improve symptoms of depression. Therefore, if a participant did not respond to the intervention by session five, they were offered two additional sessions. Estimating this indirect effect via the additional sessions involves two variables, including non-response to treatment (M3a: 0, non-response (unexposed); 1, responded to treatment (exposed)) and the number of extra sessions received in instances of non-response (M3b: 0, no extra sessions (unexposed); 1, one extra session; 2, two extra sessions). Supplementary Appendix 1 available at https://doi.org/10.1192/bjp.2022.116 details how non-response to the sessions was determined.

#### Mediator–outcome confounders

Because of the randomised nature of the exposure, it was not necessary to account for confounders for the association between the exposure and the outcome, or between the exposure and the mediators. However, it was necessary to account for confounders potentially distorting the association between the mediator and the outcome (mediator–outcome confounders). We considered all demographic characteristics as potential mediator–outcome confounders. The selection process for these confounders is described below.

### Statistical methods

#### General

To better understand the relationship between different mediators and depression outcomes, we compared characteristics of the sessions (M1a, number of sessions; M1b, proportion of homework completed), behavioural activation (M2), non-response to the intervention (M3a) and the number of extra sessions attended by participants recived who did not respond to HAP (M3b), with the outcome remission from depression (determined by a PHQ-9 score <10) for participants in the experimental arm only. Differences in baseline characteristics between treatment arms can be found in previous publications.^11,12^

#### Mediation analyses

**Fig. 1:**
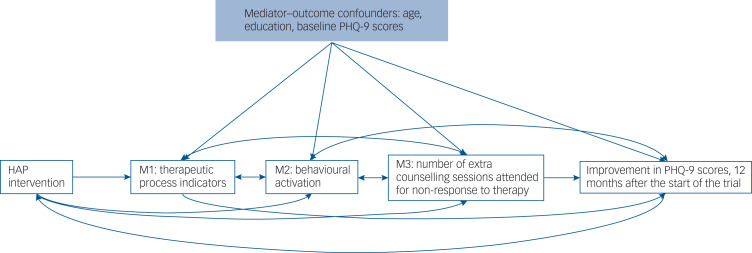
Causal model demonstrating the proposed pathways through which the HAP intervention may improve remission from depression. M1 comprised number of sessions (M1a) and proportion of homework completed (M1b); M2 comprised behavioural activation levels measured using the BAD-SF questionnaire; M3 comprised whether participants responded to treatment (M3b) and number of extra sessions a participant received in instances of non-response (M3a). HAP, Healthy Activity Program; PHQ-9, Patient Health Questionnaire.

We aimed to investigate the extent to which symptoms of depression measured at 12 months by the PHQ-9 questionnaire, were explained by indirect effects of the intervention via the above listed mediators (Fig. 1). To achieve these objectives, we used the interventional (in)direct effects approach to mediation analysis to understand population-level effects relevant to this analysis.^15^ Findings for this analyses are reported according to guidelines for reporting mediation analyses (AGReMA statement).^22^

#### Decomposition of total effect of the HAP intervention into direct and indirect effects

The first step of the mediation analyses involved decomposing the total effect of the HAP intervention into path-specific indirect effects and the direct effect. For the decomposition to be valid, the sum of the different path-specific effects and the direct effect, through which the effects of the intervention are mediated, must be the same as the total effect of the intervention.^23^

Conceptualising how the total effect of an intervention is decomposed into multiple indirect effects and the direct effect is intuitively different than approaches such as the structural equation modelling framework for a single mediator, which use multiple linear regression models for the mediator and outcome.^24,25^ With these approaches, the indirect effect for behavioural activation, for example, is the product of the coefficient in the regression of behavioural activation on the treatment arm and the coefficient of behavioural activation in the regression of the outcome on the treatment arm with behavioural activation. The code provided in Supplementary Appendix 2 details how the interventional effects method was applied to decompose the total effect of the intervention into the direct and indirect effects, in contrast to the single mediator models described above.

### Estimation and model fit

We used the estimation procedure described by Mooney,^26^ using 1000 Monte-Carlo simulations. A combination of linear (M1a, M2), ordinal categorical (M1b, M3b) and logistic regression models (M3a) were used. Models included predictors age, education, baseline PHQ-9 scores, participants expectations of treatment and marital status, which were shown to improve model fit according to the Akaike Information Criterion (AIC).^27^ These models were then used to set random, subject-specific draws for the mediator levels in the exposed and unexposed populations.

The outcome models included any mediator–outcome confounder that improved model fit according to the AIC, but were not known to be potentially influenced by them (i.e. thus excluding other mediators). The outcome models were then used to predict PHQ-9 scores at exposed and unexposed (i.e. counterfactual) levels for the different mediators.

Bias-corrected confidence intervals were based on nonparametric bootstrap with 1000 resamples.^15^ The bootstrap also accounted for clustering at the primary health clinic. Details of the estimation methods can be found in Supplementary Appendix 1 and the Stata version 17.0 (for Linux) code used to estimate mediator levels and calculate the different effects can be found in Supplementary Appendix 2.

### Assumptions

Identification of interventional effects relies on important assumptions that will influence the validity of our findings if violated. Reassuringly, because of the randomised nature of the HAP trial, many of the assumptions with the interventional effects are fulfilled. The main assumption relevant to our study is that there are no unmeasured mediator–outcome confounders. Importantly, the interventional effects capture the components of the total effect mediated by the different mediators, even when the structural dependence between multiple mediators is unknown (i.e. direction of the causal effects between the multiple mediators is unknown, or if there is unmeasured common causes of the mediators) (Fig. 1).

### Missing data

There were missing data for the BADS-SF variable (measuring M2) at 3 months (*n* = 28, 5.7%) and the PHQ-9 variable at 12 months (*n* = 47, 9.3%). To account for this, we implemented single stochastic imputation using chained equations with ten burn-in iterations, under the assumption that data was missing at random. In each of the 1000 bootstrap samples, the imputation is done once. Details of the missing data analysis can be found in Supplementary Appendix 3.

### Ethical approval and consent

The authors assert that all procedures contributing to this work comply with the ethical standards of the relevant national and institutional committees on human experimentation and with the Helsinki Declaration of 1975, as revised in 2008. All procedures involving human patients were approved by the Sangath and London School of Hygiene and Tropical Medicine (LSHTM) Institutional Review Boards (reference number 6507). Written or witnessed verbal (if the participant was illiterate) informed consent was mandatory for enrolment. All consent procedures were audio-taped, with the patient's approval, for quality assurance.

## Results

There were 493 participants included in the study, with 248 (50%) allocated to the control arm with EUC alone, and 245 (50%) to the experimental arm with EUC plus HAP. Table 1 compares mediators measured at 3 months, between participants with remission from depression (PHQ-9 score <10) and participants with depression (PHQ-9 score ≥10) measured at 12 months after the trial started, in the experimental arm only. Results suggest that participants who attended sessions (M1a) and completed more homework (M1b) were less likely to recover from depression. Findings suggest that at 12 months, participants who had sustained remission from depression had higher mean behavioural activation levels (M2) measured at 3 months, compared with participants who did not recover from depression.

**Table 1:** Comparison of mediators between participants with and without remission from depression at 12 months, in the experimental arm of the trial only

Mediators	Remission from depression, determined by PHQ-9 scores <10 at 12 months in the experimental group only (*n* = 245)^a^		Overall
	No remission from depression (*n* = 89) (PHQ-9 score >10), *n* (%)	Remission from depression (*n* = 156) (PHQ-9 score <10), *n* (%)	
Number of sessions attended			
0	6 (6.7)	13 (8.4)	19 (7.8)
1	4 (4.4)	16 (10.9)	20 (8.2)
2	7 (7.8)	17 (11.0)	24 (9.8)
3	2 (2.2)	3 (1.9)	5 (2.0)
4	2 (2.2)	2 (1.3)	4 (1.6)
5	11 (12.2)	47 (30.3)	58 (23.7)
6	23 (25.6)	31 (20.0)	54 (22.0)
7	10 (11.1)	10 (6.5)	20 (8.2)
8	25 (27.8)	16 (10.3)	41 (16.7)
M1a: number of phases of sessions completed			
None (no sessions completed)	6 (6.7)	13 (8.4)	19 (7.8)
One (sessions 1 to 2 completed)	11 (12.2)	33 (21.3)	44 (18.0)
Two (sessions 3 to 4 completed)	4 (4.4)	5 (3.2)	9 (3.7)
Three (sessions 5 to 8 completed)	69 (76.7)	104 (61.1)	173 (70.6)
M1b: proportion of assigned activities completed			
No homework	13 (14.4)	34 (21.9)	47 (19.2)
1–50%	14 (15.6)	25 (16.1)	39 (16.0)
50–100%	63 (70.0)	96 (61.9)	159 (64.9)
M2: adapted BADS-SF score measured at 3 months, mean (s.d.)	9.5 (4.8)	13.4 (4.3)	12.0 (4.7)
M3a: participant's response to HAP measured by PHQ-9 score ≥10 at third or fourth session^b^			
Did not respond to HAP	56 (62.2)	63 (40.7)	119 (48.6)
Responded to HAP	34 (37.8)	92 (59.4)	126 (51.4)
M3b: number of extra sessions attended if participant did not respond to HAP			
None	55 (61.1)	129 (83.2)	184 (75.1)
One	10 (11.1)	10 (6.5)	20 (8.2)
Two	25 (27.8)	16 (10.3)	41 (16.7)

PHQ-9, Patient Health Questionnaire; BADS-SF, Behavioural Activation for Depression Scale – Short Form; HAP, Healthy Activity Program.

a. Missing data has been imputed by trial arm, using single imputation stochastic models adjusted for any factors that could potentially influence missingness.

b. Supplementary Appendix 1 describes how non-response to counselling was determined.

### Mediation

Table 2 demonstrates that at 12 months, of the total mean difference in PHQ-9 scores between the experimental and control arms (adjusted mean difference in PHQ-9 scores: −2.1, bias-corrected 95% CI −3.2 to −1.5), 34% was mediated through indirect effects via activity levels (M2), measured with the adapted version of the BADS-SF (adjusted mean difference in PHQ-9 scores attributable to pathways via BADS-SF: −0.7, bias-corrected 95% CI −1.2 to −0.4). There was no evidence to support mediation through indirect effects via characteristics of the sessions, including number of sessions (M1a) and proportion of homework completed (M1b) (adjusted mean difference: 2.0, bias-corrected 95% CI −0.4 to 4.1). A participant's response to therapy (M3a) and the number of additional sessions received in instances of non-response (M3b) did not improve depression scores either (adjusted mean difference: −1.0, bias-corrected 95% CI −2.4 to 1.4). Findings also suggest there was no evidence that the HAP intervention improved symptoms of depression through mechanisms other than those explained with our mediators (direct effect: −2.3, bias-corrected 95% CI −4.5 to 0.6).

**Table 2:** Total effect and interventional in(direct) effects for the Healthy Activity Program intervention at 12 months

Effect	Estimates (bias-corrected 95% CI)^a^^,^^b^^,^^c^^,^^d^
Total effect	−2.1 (−3.2 to −1.5)
Interventional direct effect	−2.3 (−4.5 to 0.6)
Interventional indirect effect through attending sessions (M1a) and completing homework (M1b)	2.0 (−0.4 to 4.1)
Interventional indirect effect through behavioural activation levels (M2)	−0.7 (−1.2 to −0.4)
Interventional indirect effect through non-response to therapy (M3a) and extra sessions (M3b)	−1.0 (−2.4 to 1.4)
Interventional indirect effect through the dependency of the mediators on one another	−0.2 (−0.5 to 0.3)

a. Estimates have been adjusted for mediator–outcome confounders of baseline Patient Health Questionnaire scores, age and education.

b. Estimation for the different effects was based on Monte-Carlo integration, using a 1000-fold expanded data-set.

c. Bias-corrected confidence intervals were based on nonparametric bootstrap with 1000 resamples.

d. Missing data has been imputed by trial arm, using single imputation stochastic models adjusted for any factors that could potentially influence missingness.

## Discussion

Applying robust, interventional (in)direct effects has allowed us to understand what components of the HAP intervention are effective, and what other components may need to be revised and/or modified before considering scale-up. Specifically, our findings suggest that lay counsellors should emphasise the role of behavioural activation in improving symptoms of depression, and not necessarily focus on the number of sessions. However, if symptoms do not improve after six sessions, treatment other than offering extra sessions should be considered.

The main strategy included in the HAP intervention was behavioural activation for depression.^18^ The results from our analysis support this approach, whereby levels of behavioural activation, captured with the adapted BADS-SF, improved symptoms of depression. Our findings were also supported by a Cochrane systematic review that found behavioural activation may be more effective than medication in improving symptoms of depression.^28^ However, results from other mediation analyses evaluating the role of behavioural activation are mixed.^29^ Nevertheless, comparisons are difficult, given the different contexts, population under investigation, study designs, conditions being treated, interventions provided and methods used to conduct the analyses.

Our estimates suggest that characteristics of the sessions we had available, including the number of phases of the intervention the participant attended (M1a) and the proportion of homework completed (M1b), did not influence depression outcomes. Results from the univariate analysis can help to explain this issue, where participants who attended more sessions and completed more homework were less likely to recover from depression. Likewise, participants were less likely to recover from depression if they completed more than 50% of their homework, compared with participants who completed less homework. A likely explanation for this phenomenon is that this is a stepped-care intervention, whereby participants who did not respond to the sessions received up to two additional sessions. To address this feature in the study design, we created an additional mediator to estimate the role of the extra sessions (M3b) attended by participants who did not respond to the intervention (M3a), in improving symptoms of depression. Our findings did not indicate any benefit to receiving the extra sessions, suggesting that if the HAP intervention is brought to scale, it will be important to bear this in mind when deciding on how to improve symptoms of depression among non-responders.

Our findings indicate that a large proportion of the total effect is still unknown. These results are key, as they suggest that there were characteristics of the HAP intervention that helped to improve symptoms of depression and were not captured with the mediators that we had available. Indeed, there were four domains of strategies included as part of the sessions: engagement (psychoeducation, family psychoeducation and treatment planning), behavioural activation, need-based strategies (i.e. addressing interpersonal triggers, problem-solving, relaxation) and social integration.^18^ It is entirely conceivable that the effect of the domains not captured with the mediators that we had available was expressed through the direct effect. As an example, HAP investigators theorised that the need-based strategies that were used to address problem-solving, relaxation and enlisting social support, would improve life context, reduce life problems and eventually alleviate symptoms of depression. This issue highlights the importance of identifying and subsequently collecting relevant data on potential mediators when planning studies. This could provide greater insights into how the intervention can be optimised for future scale-up.

### Strengths and limitations

Our approach to mediation has several strengths. Importantly, our mediation analyses allowed us to include multiple mediators, their interactions and non-linearities. This is important because failing to do so could influence the strength of all the direct and indirect effects. As an example, without adjusting for characteristics of the sessions, behavioural activation could be over- or underestimated. Another strength of this approach is that it captures the components of the total effect mediated by the different mediators, even when the structural dependence between multiple mediators is unknown (i.e. direction of the causal effects between the multiple mediators is unknown, or if there is unmeasured common causes of the mediators) (Fig. 1).

When applying the interventional effects to a randomised trial, the main underlying assumption is that all important mediator–outcome confounders are accounted for. Failing to do so can potentially bias all estimates, including the direct and indirect effects. As an example, participants with depression who don't adhere to treatment are often quite different from participants with depression who do adhere to treatment, which may distort the relationships between the direct and indirect effects. Triangulating findings from a mediation analysis with other findings, such as those gathered through a qualitative component examining how context influences the acceptability, and fidelity of an intervention, will help to provide better insight into how the intervention worked.

In any mediation analysis, one of the main challenges is ensuring that the mediators are measured without error. In other words, the variables representing the mediators are capturing what was intended. The mediators in our analyses, including the number of sessions attended (M1a), homework completed (M1b), non-response to treatment (M3a) and number of extra sessions attended (M3b), were recorded by the lay health worker. It is possible that a degree of measurement error could have been introduced. Furthermore, the number of sessions attended and homework completed serve as only a proxy for the ‘dose’ of the intervention.

Findings of our mediation analyses need to be interpreted with caution when generalising to different contexts, such as those in African countries. However, by conducting robust mediation analyses for similar interventions in different contexts, we will be able to better understand what components of the intervention worked for whom and under what circumstances.

To conclude, this paper uses a robust approach to mediation analysis to understand how complex psychological therapies work and under what circumstances, which can potentially be used to inform policy.^15,30^ Not only do our findings reinforce the importance of emphasising behavioural activation found with other mediation analyses,^12,14^ but they also imply that, in the context of the HAP trial, the number of sessions attended and the amount of homework completed is not necessary indicative of an improvement in symptoms of depression. Moreover, estimates suggest that if participants do not respond to the intervention, attending an additional one to two sessions does not improve symptoms of depression. More research is needed to understand how interventions such as the HAP trial can be adapted to improve outcomes in non-responders.

## Data Availability

The data that support the findings of this study are openly available at the LSHTM Data Compass Platform: https://datacompass.lshtm.ac.uk/id/eprint/513/.

## Supplementary material

For supplementary material accompanying this paper visit https://doi.org/10.1192/bjp.2022.116. click here to view supplementary material
