# Supplementary material for: Understanding the effects of a complex psychological intervention on symptoms of depression in Goa, India: findings from a causal mediation analysis
Source: Br J Psychiatry. 2023 Feb;222(2):67–73. doi: 10.1192/bjp.2022.116 (PMC10895505; doi:10.1192/bjp.2022.116)
Supplement: Supplementary file 1 [file S0007125022001167sup.zip › S0007125022001167sup001.docx]

**Appendix 1 – supplementary statistical methods**

Assessing for whether a participant was not responding to treatment and therefore would be offered more sessions.

It is possible, some of the effect of the HAP intervention was mediated through the extra sessions offered to some of the participants if they were not responding to the sessions. Estimating this indirect effect requires an additional mediator, measured using two variables: whether a participant responded to the sessions (M3a), and: the number of extra sessions received (M3b).

*M3a: non-response to sessions*

From the second session onwards, participants were assessed for their response to the intervention using the PHQ-9 questionnaire. According to the HAP program manual, a participant is considered to not be responding and offered additional sessions if they had a PHQ-9 score greater than nine at sessions three or four.([25](#_ENREF_25)) Additionally, if a participant scores positive for question 9 on the PHQ-9 questionnaire (question assessing suicide risk), additional sessions were offered. Capturing non-response to the intervention was not straight forward, given not all participants completed the sessions as intended (i.e. dropped out before session five) for reasons that are unknown. Additionally, only the total in-session PHQ-9 scores were available and therefore it was not possible to know if a participant indicated a suicide risk when completing question nine of the questionnaire. Supplementary table 1 describes how non-response to sessions was determined.

**Table 1: Criteria used to determine whether a participant responded to HAP**

| **Number of sessions completed** | **Non-response** | **Response** | **Justification** |
| --- | --- | --- | --- |
| 0 or 1 session | All participants considered as not responding to HAP | Not relevant | It would not be possible for the intervention to elicit a therapeutic effect by the end of the first session which is why the PHQ-9 was only assessed after this time. |
| 2 – 4 sessions | Any participant with a PHQ-9 score taken at any session that was greater than 9 | Participants with all available in-session PHQ-9 scores less than 10 | If a participant had a PHQ score greater than 9, it is assumed they dropped out of the trial as they did not feel they were responding to treatment.  Likewise, if a participant had a PHQ-9 score less than 10 it is assumed they were feeling better and stopped attending sessions. |
| 5 - 8 sessions | Any participant with a PHQ-9 score taken in-sessions 3-6 that was greater than 9 | Participants with all available PHQ-9 scores less than 10 at sessions 3-6 | A participant who had a PHQ-9 score greater than 9 at the second session, but PHQ-9 scores less than 10 at sessions 3-6, were likely responding to treatment.  A participant who had a PHQ-9 score less than 9 at sessions 3-4, but greater than 9 at sessions 5-6 were likely not responding and/or at risk of suicide. |

Estimation methods and model fit

We used the estimation procedure described in the reference using 1,000 Monte-Carlo simulations.([24](#_ENREF_24)) A combination of linear (M1a, M2), ordinal (M1b, M3a) and logistic regression models (M3b) were used for the different mediators. Predictors were included in these models if they improved the Akaike Information Criterion (AIC). ([25](#_ENREF_25)) Any non-linearities of continuous variables and interactions between mediators or mediator and confounders, were included in the model if they were significant at the 5% level, using Stata’s post-estimation *testparm* command. Using these criteria, these models used a combination of predictors including age, education, baseline PHQ-9 scores, participants expectations of treatment, and marital status.

These models were then used to set random, subject-specific draws for the mediator levels in the exposed and unexposed populations. This ensured that mediator values drawn were more specific to the considered individual, thereby providing better insight into mechanism.

When the mediator was measured in both arms of the trial, the exposed status was taken from participants in the experimental arm and the unexposed status was taken from participants in the control arm in the trial. However, this was not possible for mediators that were part of the HAP intervention (i.e. characteristics of the sessions) and therefore not measured in the control arm. In these instances, the unexposed status was set to zero (i.e. representing no sessions were attended as was the case for a small number of participants in the experimental arm).

The total, direct and indirect effects (outcome models), used similar methods to build the mediator models, except instead of including predictors, we included any mediator-outcome confounder that improved model fit, but not known to be potentially influenced by them (i.e., thus excluding outcome, and other mediators for instance). The outcome models were then used to predict PHQ-9 scores at exposed and unexposed (i.e. counterfactual) levels for the different mediators.

Normality assumptions for all linear regression models were evaluated by examining the residual plots after running the regression commands using the post-estimation command *predict r, resid* followed by the *pnorm* and *qnorm* commands. Bias-corrected confidence intervals were based on nonparametric bootstrap with 1,000 resamples.([15](#_ENREF_15)) The bootstrap also accounted for clustering at the primary health clinic. Details of the Stata code used to estimate mediator levels and calculate the different effects can be found in Appendix 2.
