# Supplementary material for: Understanding the effects of a complex psychological intervention on symptoms of depression in Goa, India: findings from a causal mediation analysis
Source: Br J Psychiatry. 2023 Feb;222(2):67–73. doi: 10.1192/bjp.2022.116 (PMC10895505; doi:10.1192/bjp.2022.116)
Supplement: Supplementary file 1 [file S0007125022001167sup.zip › S0007125022001167sup003.docx]

**APPENDIX 2**

**Variables used in the programme:**

**Outcome**

y: PHQ-9 score (discrete)

**Exposure**

x: trial arm (binary)

**Mediators:**

Med1a: sessions (discrete)

Med1b: homework (categorical)

Med2: levels of behavioural activation (discrete)

Med3a: number of extra sessions (categorical)

Med3b: non-response to therapy (binary)

**Confounders:**

C1: age (discrete)

C2: education (categorical)

C3: baseline PHQ-9 score (discrete)

**Interactions:**

non-response to therapy*age: Med3b_C1

baseline PHQ-9 score*education: C2_C3

**Variables to predict missingness and to set mediators:**

miss_1: gender (binary)

miss_2: married (binary)

miss_3: expectations of treatment (discrete)

miss_4: job (binary)

miss_5: stigma (binary

miss_6: age group (categorical)

*The program HAP, that performs the imputation and then the Monte

*Carlo simulations, is listed below:

*******************************************************************************************

cap program drop HAP

program define HAP, rclass

set seed 1234

******************************************************************************

*Imputation for missing data

******************************************************************************

qui{

foreach var in Med2 Y {

gen imp_`var'=`var'

}

foreach var in Med2 Y {

summ `var' if x==1

replace imp_`var'=r(mean) if imp_`var'==. & x==1

}

foreach var in Med2 Y {

summ `var' if x==0

replace imp_`var'=r(mean) if imp_`var'==. & x==0

}

forvalues cycle=1(1)10 {

**** for M2

regress Med2 C3 i.miss_6 i.C2 i.miss_1 i.miss_2 i.miss_3 i.miss_4 if x==1

predict ba_1 if x==1

replace imp_Med2=ba_1 + e(rmse)*rnormal() if Med2==. & x==1

drop ba_1

regress Med2 C3 i.miss_6 i.C2 i.miss_1 i.miss_2 i.miss_4 i.miss_3 if x==0

predict ba_0 if x==0

replace imp_Med2=ba_0 + e(rmse)*rnormal() if Med2==. & x==0

drop ba_0

*FOR THE OUTCOME

regress Y i.miss_6 i.C2 C3 C2_C3 i.miss_2 i.miss_1 i.miss_3 i.miss_4 if x==1

predict p12_1 if x==1

replace imp_Y=p12_1 + e(rmse)*rnormal() if Y==. & x==1

drop p12_1

regress Y i.miss_6 i.C2 C3 C2_C3 i.miss_2 i.miss_1 i.miss_3 i.miss_4 if x==0

predict p12_0 if x==0

replace imp_Y=p12_0 + e(rmse)*rnormal() if Y==. & x==0

drop p12_0

}

replace Y=imp_Y if Y==.

replace Med2=imp_Med2 if Med2==.

drop imp_*

}

******************************************************************************

*MONTE CARLO STEP

******************************************************************************

cap program drop med_complex

program define med_complex, rclass

set seed 123985

cap drop E_*

cap drop D_*

cap drop IIE_M1*

cap drop IIE_M2*

cap drop IIE_M3*

cap drop IIE_1

cap drop IIE_2

cap drop IIE_3

cap drop IIE_123

cap drop IDE

cap drop TCE

cap drop original

cap drop M1a

cap drop M1b

cap drop M2_1

cap drop M2_0

cap drop M3a

cap drop M3b

expand 1000

sort B_PID

qui by B_PID:gen original=_n==1

****Setting mediators

**** M1 characteristics of sessions

****M1a sessions in exposed (unexposed=0)

regress med1a C1 i.C2 C3 i.miss_1 if x==1 & original==1

qui gen A=rnormal()

gen m1a=_b[_cons]+_b[C1]*C1+_b[C3]*C3+_b[1.C2]*C2+_b[1.miss_1]*miss_1+e(rmse)*A

sum m1a

drop A

*M1b homework in exposed (unexposed=0)

ologit med1b C1 i.C2 i.miss_5 i.miss_1 if x==1 & original==1

predict p1 p2 p3 , p

generate u1 = runiform()

gen M1b=.

replace M1b=2 if u1<p1+p2+p3

replace M1b=1 if u1<p1+p2

replace M1b=0 if u1<p1

sum M1b

drop p1 p2 p3 u1

****M2 - behavioural activation levels

* exposed

regress Med2 i.miss_2 i.miss_5 if x==1 & original==1

qui gen B1=rnormal()

gen M2_1=_b[_cons]+_b[1.miss_2]*miss_2+_b[1.miss_5]*miss_5+e(rmse)*B1

sum M2_1

drop B1

*unexposed

regress Med2 C1 i.miss_5 if x==0 & original==1

qui gen B0=rnormal()

gen M2_0=_b[_cons]+_b[C1]*C1+_b[1.miss_5]*miss_5+e(rmse)*B0

sum M2_0

drop B0

**** M3 extra sessions for non response to therapy

****M3a – extra sessions

* exposed (unexposed==0)

ologit Med3a C1 C3 i.miss_3 if x==1 & original==1

predict p1 p2 p3, p

generate u1 = runiform()

gen M3a=.

replace M3a=2 if u1<p1+p2+p3

replace M3a=1 if u1<p1+p2

replace M3a=0 if u1<p1

sum M3a

drop p1 p2 p3 u1

****M3b – non response to therapy

* exposed (unexposed==0)

logistic Med3b i.miss_3 if x==1 & original==1

gen M3b=runiform()<1/(1+exp(-(_b[_cons]+_b[1.miss_3]*miss_3)))

sum M3b

***** Outcome models for Total effect, direct effect, indirect effect

***** E{Y(1)} *****

*** total effect exposed

syntax [varlist] [if]

save temp, replace

keep if x==1 & original==1

regress Y C1 i.C2 C3 C2_C3 `if'

use temp, clear

predict E_1

sum E_1 `if'

local E_1=r(mean)

**** total effect, unexposed

use temp, clear

keep if x==0 & original==1

regress Y C1 i.C2 C3 C2_C3 `if'

use temp, clear

predict E_0

summ E_0 `if'

local E_0=r(mean)

** DIRECT EFFECTS

*Calculating D_1, exposed

use temp, clear

keep if x==1 & original==1

regress Y Med1a i.Med1b Med2 i.Med3a i.Med3b Med3a_C1 C1 i.C2 C3 C2_C3 `if'

use temp, clear

gen Med1a_new=Med1a

replace Med1a=0

gen Med1b_new=Med1b

replace Med1b=0

gen Med2_new=Med2

replace M2=M2_0

gen Med3b_new=Med3b

replace Med3b=0

gen Med3b_C1_new=Med3b_C1

replace Med3b_C1=0

gen Med3a_new=Med3a

replace Med3a=0

predict D_1

summ D_1 `if'

local D_1=r(mean)

replace Med1a=Med1a_new

drop Med1a_new

replace Med1b=Med1b_new

drop Med1b_new

replace Med2=Med2_new

drop Med2_new

replace Med3b=Med3b_new

drop Med3b_new

replace Med3b_C1=Med3b_C1_new

drop Med3b_C1_new

replace Med3a=Med3a_new

drop Med3a_new

** direct effect, unexposed

*Calculating D_0

use temp, clear

keep if x==0 & original==1

regress Y C1 i.C2 C3 C2_C3 Med2 `if'

use temp, clear

gen Med2_new=Med2

replace M2=M2_0

predict D_0

summ D_0 `if'

local D_0 =r(mean)

replace Med2=Med2_new

drop Med2_new

* Intervention indirect effect (IIE_M1) for M1

*M1 at levels in the exposed

use temp, clear

keep if x==1 & original==1

regress Y Med1a i.Med1b Med2 i.Med3a i.Med3b Med3a_C1 C1 i.C2 C3 C2_C3 `if'

use temp, clear

gen Med1a_new=Med1a

replace Med1a=M1a

gen Med1b_new=Med1b

replace Med1b=M1b

gen Med2_new=Med2

replace Med2=M2_0

gen Med3b_new=Med3b

replace Med3b=0

gen Med3b_C1_new=Med3b_C1

replace Med3b_C1=0

gen Med3a_new=Med3a

replace Med3a=0

predict IIE_M1_1

summ IIE_M1_1 `if'

local IIE_M1_1=r(mean)

replace Med1a=Med1a_new

drop Med1a_new

replace Med1b=Med1b_new

drop Med1b_new

replace Med2=Med2_new

drop Med2_new

replace Med3b=Med3b_new

drop Med3b_new

replace Med3b_C1=Med3b_C1_new

drop Med3b_C1_new

replace Med3a=Med3a_new

drop Med3a_new

***M1 at levels in the unexposed

use temp, clear

keep if x==1 & original==1

regress Y Med1a i.Med1b Med2 i.Med3a i.Med3b Med3a_C1 C1 i.C2 C3 C2_C3 `if'

use temp, clear

gen Med1a_new=Med1a

replace Med1a=0

gen Med1b_new=Med1b

replace Med1b=0

gen Med2_new=Med2

replace Med2=M2_0

gen Med3b_new=Med3b

replace Med3b=0

gen Med3b_C1_new=Med3b_C1

replace Med3b_C1=0

gen Med3a_new=Med3a

replace Med3a=0

predict IIE_M1_0

summ IIE_M1_0 `if'

local IIE_M1_0=r(mean)

replace Med1a=Med1a_new

drop Med1a_new

replace Med1b=Med1b_new

drop Med1b_new

replace Med2=Med2_new

drop Med2_new

replace Med3b=Med3b_new

drop Med3b_new

replace Med3b_C1=Med3b_C1_new

drop Med3b_C1_new

replace Med3a=Med3a_new

drop Med3a_new

**** Calculate IIE_2

*** M2 at levels in the exposed

use temp, clear

keep if x==1 & original==1

regress Y Med1a i.Med1b Med2 i.Med3a i.Med3b Med3a_C1 C1 i.C2 C3 C2_C3 `if'

use temp, clear

gen Med1a_new=Med1a

replace Med1a=M1a

gen Med1b_new=Med1b

replace Med1b=M1b

gen Med2_new=Med2

replace Med2=M2_1

gen Med3b_new=Med3b

replace Med3b=0

gen Med3b_C1_new=Med3b_C1

replace Med3b_C1=0

gen Med3a_new=Med3a

replace Med3a=0

predict IIE_M2_1

summ IIE_M2_1 `if'

local IIE_M2_1=r(mean)

replace Med1a=Med1a_new

drop Med1a_new

replace Med1b=Med1b_new

drop Med1b_new

replace Med2=Med2_new

drop Med2_new

replace Med3b=Med3b_new

drop Med3b_new

replace Med3b_C1=Med3b_C1_new

drop Med3b_C1_new

replace Med3a=Med3a_new

drop Med3a_new

** M2 at levels in the unexposed

use temp, clear

keep if x==1 & original==1

regress Y Med1a i.Med1b Med2 i.Med3a i.Med3b Med3a_C1 C1 i.C2 C3 C2_C3 `if'

use temp, clear

gen Med1a_new=Med1a

replace Med1a=M1a

gen Med1b_new=Med1b

replace Med1b=M1b

gen Med2_new=Med2

replace M2=M2_0

gen Med3b_new=Med3b

replace Med3b=0

gen Med3b_C1_new=Med3b_C1

replace Med3b_C1=0

gen Med3a_new=Med3a

replace Med3a=0

predict IIE_M2_0

summ IIE_M2_0 `if'

local IIE_M2_0=r(mean)

replace Med1a=Med1a_new

drop Med1a_new

replace Med1b=Med1b_new

drop Med1b_new

replace Med2=Med2_new

drop Med2_new

replace Med3b=Med3b_new

drop Med3b_new

replace Med3b_C1=Med3b_C1_new

drop Med3b_C1_new

replace Med3a=Med3a_new

drop Med3a_new

****** Calculate IIE_3**

*** M3 at levels in the exposed

use temp, clear

keep if x==1 & original==1

regress Y Med1a i.Med1b Med2 i.Med3a i.Med3b Med3a_C1 C1 i.C2 C3 C2_C3 `if'

use temp, clear

gen Med1a_new=Med1a

replace Med1a=M1a

gen Med1b_new=Med1b

replace Med1b=M1b

gen Med2_new=Med2

replace Med2=M2_1

gen Med3b_new=Med3b

replace Med3b=M3b

gen Med3b_C1_new=Med3b_C1

replace Med3b_C1=M3*C1

gen Med3a_new=Med3a

replace Med3a=M3a

predict IIE_M3_1

summ IIE_M3_1 `if'

local IIE_M3_1=r(mean)

replace Med1a=Med1a_new

drop Med1a_new

replace Med1b=Med1b_new

drop Med1b_new

replace Med2=Med2_new

drop Med2_new

replace Med3b=Med3b_new

drop Med3b_new

replace Med3b_C1=Med3b_C1_new

drop Med3b_C1_new

replace Med3a=Med3a_new

drop Med3a_new

** M3 at levels in the unexposed

use temp, clear

keep if x==1 & original==1

regress Y Med1a i.Med1b Med2 i.Med3a i.Med3b Med3a_C1 C1 i.C2 C3 C2_C3 `if'

use temp, clear

gen Med1a_new=Med1a

replace Med1a=M1a

gen Med1b_new=Med1b

replace Med1b=M1b

gen Med2_new=Med2

replace Med2=M2_1

gen Med3b_new=Med3b

replace Med3b=0

gen Med3b_C1_new=Med3b_C1

replace Med3b_C1=0

gen Med3a_new=Med3a

replace Med3a=0

predict IIE_M3_0

summ IIE_M3_0 `if'

local IIE_M3_0=r(mean)

replace Med2=Med2_new

drop Med2_new

replace Med1a=Med1a_new

drop Med1a_new

replace Med1b=Med1b_new

drop Med1b_new

replace Med3b=Med3b_new

drop Med3b_new

replace Med3b_C1=Med3b_C1_new

drop Med3b_C1_new

replace Med3a_new=Med3a

drop Med3a_new

*** RESULTS

local IIE_3 = `IIE_M3_1' - `IIE_M3_0'

local IIE_2 = `IIE_M2_1' - `IIE_M2_0'

local IIE_1 = `IIE_M1_1' - `IIE_M1_0'

local IDE = `D_1' - `D_0'

local TCE = `E_1' - `E_0'

local IIE_123 = ((`TCE')-((`IIE_3')+(`IIE_2')+(`IIE_1')+(`IDE')))

return scalar IIE_3 = `IIE_3'

return scalar IIE_2 = `IIE_2'

return scalar IIE_1 = `IIE_1'

return scalar IDE = `IDE'

return scalar TCE = `TCE'

return scalar IIE_123 = `IIE_123'

end

bootstrap r(IIE_3) r(IIE_2) r(IIE_1) r(IIE_123) r(IDE) r(TCE), noi reps(1000) cluster (clinic) idcluster(clin) seed (1234): HAP

estat bootstrap, all

ex
